# Upcycling Discarded Shoe Polish into High Value-Added Asphalt Fluxing Agent for Use in Hot Mix Paving Applications

**DOI:** 10.3390/ma15186454

**Published:** 2022-09-17

**Authors:** Nader Nciri, Namho Kim

**Affiliations:** 1 School of Industrial Design & Architectural Engineering, Korea University of Technology & Education, 1600 Chungjeol-ro, Byeongcheon-myeon, Dongnam-gu, Cheonan 31253, Chungnam, Korea; 2 School of Energy, Materials & Chemical Engineering, Korea University of Technology & Education, 1600 Chungjeol-ro, Byeongcheon-myeon, Dongnam-gu, Cheonan 31253, Chungnam, Korea

**Keywords:** hot mix asphalt, discarded shoe polish, fluxing agent, TLC-FID, FT-IR, empirical tests, flash point, DSR, MSCR, BBR

## Abstract

This research effort is geared towards revealing the latent potential of discarded shoe polish that might be repurposed as an asphalt fluxing agent for the construction of durable and sustainable road surfaces. To drive this creative invention, the effect of various proportions of waste shoe polish (e.g., 5, 10 and 15 wt. % WSP) on the performance of base AP-5 bitumen was inspected in great detail. A meticulous investigation of the chemical, physical, and rheological properties of the resultant combinations was carried out using a variety of state-of-the-art laboratory techniques, specifically: thin-layer chromatography-flame ionization detection (TLC-FID), Fourier transform-infrared spectroscopy (FT-IR), needle penetration, ring-and-ball softening point, Brookfield viscometer, ductility, flash/fire points, dynamic shear rheometer (DSR), multiple stress-creep recovery (MSCR), and bending beam rheometer (BBR) tests. The Iatroscan data disclosed that the continuous feeding of binder with WSP had a minor impact on SARA fractional distribution, regardless of aging. According to the FT-IR scan, the stepwise addition of WSP to the binder did not result in any significant chemical alterations in the blends. The combined outcomes of the DSR/BBR/empirical test methods forecasted that the partly bio-sourced additive would greatly improve the mixing–compaction temperatures, workability, and coating–adhesion properties of bituminous mixtures while imparting them with outstanding anti-aging/cracking attributes. In short, the utilization of waste shoe polish as a fluxing agent for hot asphalt mix production and application is not only safe, feasible, and affordable, but it has the potential to abate the pollution caused by the shoe-care market while simultaneously enhancing the overall performance of the pavement and extending its service lifespan.

## 1. Introduction

The lifespan of paved roadways is chiefly dependent upon certain definite factors; some of them are external and some others are internal. The external factors that may affect the performance of a given pavement cover mechanical (i.e., traffic loading) and climatic factors such as air, sunlight, heat, water, and others, whereas the internal factors entail the composition of the entire block constituting the road pavement, such as rocky aggregates (e.g., crushed gravel/stone, 85 wt. %), gummy binder (i.e., glue, 10 wt. %), and voids (5 wt. %), etc. [1].

Owing to its vital role that it plays in sustaining the entire structure and function of bituminous concrete surfaces, asphalt cement is regarded as one of the main key components that governs both the bearing capacity and durability of a given roadway. Over time, the binder steadily ages and loses its engineering assets, resulting in several dramatic issues ranging from permanent deformation (e.g., corrugations, rutting, shoving) to cracking (e.g., reflective, thermal, fatigue), etc. [2].

In order to circumvent such distresses, some bio-based or artificial additives or modifiers have been created aimed at empowering or repairing the damaged bitumen properties in terms of adhesion, elasticity, fluidity, oxidation features, or skid resistance, etc.

Additives may comprise fillers, fibers, powders, waxes, emulsifiers, as well as recycling agents. The recycling agents are hydrocarbon-based products with specific physicochemical features, and they are used specifically to rehabilitate the chemical, physical, and rheological properties of aged asphalt binders [3].

Two main categories exist, namely, rejuvenating agents and softening agents [4]. The rejuvenating agents, such as extender oils and lube extracts, which possess a high concentration of maltenes components (i.e., saturates, aromatics and resins), may assist in restoring the maltene-to-asphaltene ratio that has been altered during the aging process. Therefore, the employment of rejuvenators may greatly boost the ductility, relaxation, and adhesive–cohesive attributes of reclaimed binders [4]. Meanwhile, the softening agents, including slurry oils, crankcase oils, lubricating oils, and lube stocks, are relatively non-volatile crude oil fractions with an appropriate viscosity, and they are specifically created with the aim of solely lowering the viscosity of the aged asphalt cement [5].

The softeners may comprise also volatile petroleum solvents, such as gasoline, naphtha, kerosene, diesel fuel, and mineral spirits, etc., commonly known as flux oils/cutback agents/cutters, which are specifically used to momentarily reduce (i.e., cutback) the bitumen viscosity, allowing for easier handling and processing at lower temperatures (i.e., lower energy/carbon emissions), especially in the production of cold-mix (patching) asphalt [6].

For instance, it has been reported that kerosene (medium curing), diesel (slow curing), and lubricating oil are classified among the finest cutters that can generate high-quality cutback bitumina for priming road surfaces. Indeed, these lighter petroleum products, or diluents, were discovered to be capable of decreasing the consistency as well as the viscosity of blends, thereby lowering the mixing temperature [7].

Furthermore, it has been found that adding petroleum distillates, notably mineral spirits, can improve the workability, coatability, resistance to rutting and fatigue cracking, moisture stability, and curing of cold-asphalt mixes [8].

On the other side, some plant-based (vegetal) fluxes such as rapeseed and linseed oil methyl esters can drastically lower the temperature needed for mixing, laying, and compacting asphalt–aggregate mix [9]. Meanwhile, certain semi-volatile bio-sourced solvents such as hydrophilic di-methyl isosorbide ethers (DMI) have shown promise in temporarily reducing the Newtonian viscosity of asphalt mixes, hence facilitating their manipulation during wet seasons [10].

The partnership of Solvay with Eurovia has fruitfully resulted in the creation of a breakthrough bio-based fluxing agent dubbed “InnRoad Protect^TM^” for the road and related industries. When compared to conventional fluxing agents, “InnProtect”, which has an outstanding HSE (i.e., hygiene, safety and environment) profile, can deliver superior fluxing power, quicker cohesion build-up, improved bitumen recovery qualities, and can also prolong the maintenance season [6].

As part of the worldwide movement to tackle pollution, numerous international highway agencies and state transportation departments have attempted to recycle and repurpose a broad spectrum of materials and by-products into multiple road construction projects such as plastic [11], ground rubber tires [12], cardboard [13], glass [14], construction and demolition waste [15], asphalt shingles [16], reclaimed asphalt pavement [17], incinerator ash [18], and so forth. By utilizing these salvaged ingredients, not only the performance and serviceability of road pavement could be boosted, but also enormous quantities of non-renewable natural resources could be preserved, massive volumes of emitted green gas and air pollutants could be shrunk, an extensive area of land could be spared from being dumped, and, most importantly, a healthy and well-balanced ecosystem could be established.

Research and trials into novel and cutting-edge applications of waste materials are continuously making strides forward. Waste cooking oil [19], waste animal fats [20], discarded chewing gums [21], leftover lipsticks [22], expanded polystyrene waste [23], and so on are among the household and industrial waste items that have been assessed by our professionals and beneficially incorporated into several sustainable road infrastructure projects.

By unceasingly digging deeper into the accessible waste streams, it is possible to discover a plethora of yet untapped potential compounds from diverse origins that could effectively be integrated within the transportation area. A concrete example of this is the unusable, unwanted, and leftover shoe polish.

Shoe polish (SP, also known as boot polish) can be found in different forms, either as a cream emulsion or liquid, or even as a waxy paste, and can be applied using a brush, rag, or cloth. SP is deliberately designed to prolong the lifetime and restore the beauty of boots and leather shoes by replenishing the fats and oils that have been lost from them and ensuring their proper waterproof protection. Indeed, SP helps to get rid of coarse wrinkles, cracks, scratches, scuffs, and color abrasions from leather footwear [24,25].

Most SPs are made out of a variety of intriguing ingredients such as organic solvents (e.g., petroleum distillate–naphtha, Stoddard solution, oleic acid, C13–14 alkanes and 1-chloro-4(trifluoromethyl)-benzene, etc.; ~70 wt. %), natural or synthetic waxes (e.g., beeswax, lanolin/wool wax, montan/lignite/OP wax such as montan acid wax or glycol montanate, carnauba/Brazil/palm wax, paraffin and microcrystalline waxes, and polyethylene wax; 20~40 wt. %), oils (e.g., turpentine oil, olive oil, palm oil and mink oil, etc.), gums (e.g., gum Arabic), alcohols (e.g., ethylene glycol), and colorants (e.g., pigments or soluble dyes viz. C.I. 50415:1 (black) and C.I. 11021 (yellow); 2~3 wt. %) [25,26,27].

The waste shoe polish (WSP) winds usually up in landfills or dumps and pollutes the environment by releasing toxic chemicals into the soil, groundwater, and atmosphere. Dyes liberate hazardous heavy metals, while blocky waxes or liquid emulsions disintegrate into oily residues, petroleum-based substances, and noxious volatile organic compounds (VOCs) [28,29].

Some of these substances (e.g., nitrobenzene, aniline, tar products, lanolin and isopropyl alcohol, etc.) are proven carcinogens and have devastating impacts on human and animal bodies, whether they are inhaled, ingested, or absorbed through the skin [30,31,32]. Short-term and long-term exposure to trace amounts of these chemicals may trigger carcinogenesis via their direct reaction with the DNA, causing mutations and, ultimately, initiating abnormal cellular proliferation. For example, certain dyes may cause lung cancer if regularly inhaled. Ingestion of nitrates or benzenes over time may also induce stomach cancer. The exposure to such substances is very hazardous [30,31,32].

The global shoe polish market is exponentially growing on the back of growing sales and demand for fashionable and comfortable footwear across the world [33]. This has led in turn to an exacerbated pollution burden caused by discarded shoe polish that can severely threaten public health and planetary health [31,34].

To take bold and inventive steps towards curbing the shoe polish pollution crisis while achieving long-term pavement performance as well as contributing to a circular economy, this preliminary investigation was undertaken. Its primary goal was to divert a relatively green WSP, which is rich in oleochemical esters and petrochemical solvents, into a viscosity-reducing agent or asphalt fluxing agent for use in hot-mix paving-related applications.

## 2. Materials and Methods

### 2.1. Preparation of Waste Shoe Polish (WSP)–Asphalt Blends

The base AP-5 asphalt (PG 70–22) employed in this investigation was graciously supplied by the South Korean Federation of Ascon Industry Cooperative R&D Center (Osan-si, Gyeonggi-do, South Korea). Table 1 summarizes its typical physicochemical properties.

**Figure 1:**
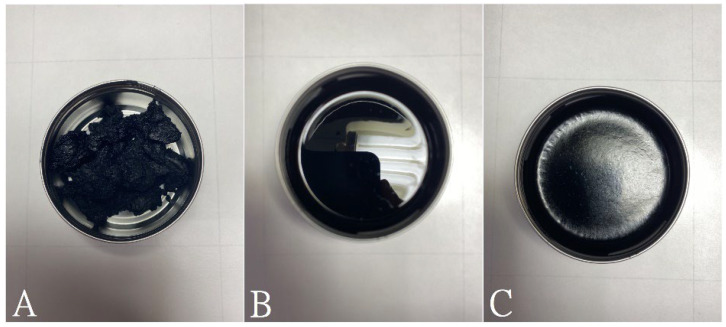
Leftover/waste shoe polish (WSP) specimens in different states: (**A**) Waxy solid paste, (**B**) Liquid emulsion formed after heating the waxy solid paste in the oven for 15 min at 100 °C, (**C**) Rigid waxy paste formed after cooling and solidifying the liquid emulsion at room temperature (ca. 25 °C).

Figure 1 depicts leftover or waste shoe polish (WSP) that was directly recovered from a household hazardous disposal site located in Cheonan-city, Chungnam-province, South Korea. This petroleum-based item, which must not be placed in the trash, is usually picked up by the local authorities, via door-to-door or curbside collection rounds from residents and businesses, or it is delivered straight to a household hazardous waste facility. The end-of-waste criteria, set by South Korea’s Waste Management Law, define when discarded shoe polish ceases to be waste or turns into a finished product or secondary raw material, in particular if it is commonly used for specific applications, or it is in great demand, or it has a well-established market, or even if it possesses higher potential to be repurposed as an asphalt additive for the road paving industry. Among the other criteria to consider is that the waste in question should not pose any adverse impact on the environment or human health.

**Figure 2:**
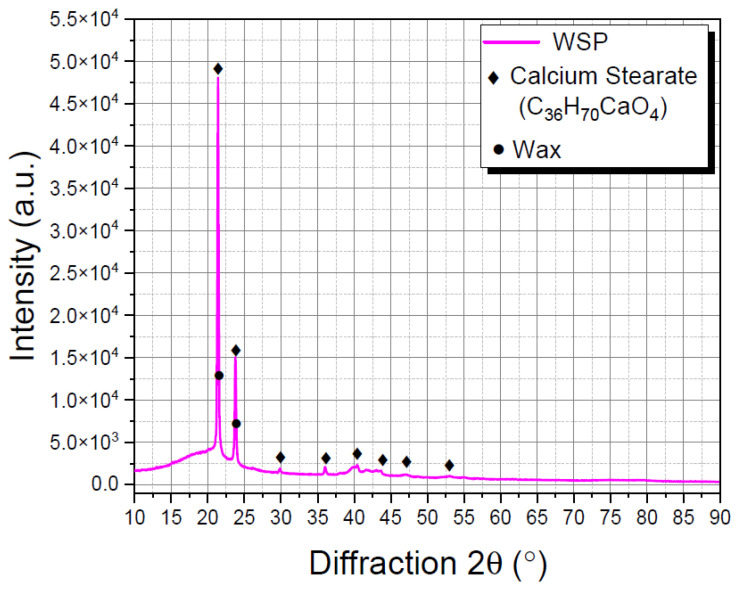
XRD diffractogram of waste shoe polish (WSP).

The basic formula of WSP is synthesized in Table 2, whereas its chemical and physical characteristics are recapitulated in Table 1. As seen from Table 2, shoe polish is made up of a variety of natural and synthetic ingredients, ranging from solvents to waxes, plasticizers, texturizing agents, dyes, and pigments. The XRD diffractogram of WSP, shown in Figure 2, displays two distinctive sharp peaks at 2θ of 21.40° and 23.78°, which correspond to the diffractions of (110) and (200) crystal planes of wax, respectively [35]. Meanwhile, calcium stearate (C_36_H_70_CaO_4_) is linked to a cluster of major and minor peaks scattered across the 20~55° region [36].

The preparation of WSP–AP-5 blends was accomplished by means of a heating mantle (Model No. GLHMD-B100, Global Lab Co., Ltd., Siheung-si, Gyeonggi-do, South Korea) set at 180 °C and a L5M-A high-shear laboratory mixer (Model No. GLHMD-B100, Silverson, East Longmeadow, MA, USA) operating at 3000 rpm [37,38]. To obtain adequate liquidity/workability with less oxidation impact, the original binder was placed in the oven and heated up for 2 h from room temperature (ca. 25 °C) to 140 °C. Afterwards, a 1000-mL cylindrical aluminum can was gently filled with 600 g of fluid asphalt and then subjected to heat-conditioning from 140 to 175 °C. Upon reaching 175 °C, various fractions of WSP (e.g., 5, 10, and 15 wt. % WSP by combination’s overall weight) in the form of waxy solid paste (Figure 1A) were stepwise added to the plain binder. The different additive concentrations were intentionally selected with the aim of effectively and efficiently assessing the performance of asphaltic admixtures based on their physicochemical and rheological attributes. To simulate conventional blending procedures, the several homogenous dispersions were obtained by applying a steady mix at 3000 rpm for 2 hours at 180 °C [37,38]. Lastly, the mixtures were kept in metal tins with airtight lids until they were ready to be artificially aged and tested.

Adding to the fact that the bulk of the WSP originates mainly from the oleochemical division’s fatty acids (FAs) (e.g., primarily oleic acid (FA) with some amount of Carnauba wax (FA), Montan acid wax (FA), and glycol montanate (FA)), the current mixing protocol was adopted since previous studies [37,38] demonstrated its effectiveness in the creation of stable bio-fluxed bitumina with promising engineering features.

When the fluxing agent was slowly added to the base AP-5 asphalt, there was an instantaneous generation of fumes and vapors, resulting chiefly from the combustion of WSP’s organic compounds. Depending on the additive dosage, the volume of emitted gas lasted for a few minutes before vanishing considerably with mixing time.

Due to the fact that the plain AP-5 asphalt also released some smoke while mixing, it is quite challenging to ascertain whether the modification caused more fumes or not; and, more particularly, the fume levels produced by both unmodified and modified binders (over two hours) are virtually comparable and pretty negligible.

Therefore, instead of relying solely on visual monitoring of gas emissions, it would be ideal to employ some advanced analytical techniques, such as gas-liquid chromatography coupled to mass spectroscopy (GC/MS) or high-performance liquid chromatography (HPLC), to identify and quantify the various air pollutants (e.g., volatile organic compounds (VOCs), polycyclic aromatic hydrocarbons (PAHs), particulates, carbon monoxide (CO), carbon dioxide (CO_2_), nitrogen oxides (NO_x_) and sulfur oxides (SO_x_), etc.) present in the fume [39]. This would tremendously assist in better defining the HSE (i.e., hygiene, safety and environment) profile of WSP.

### 2.2. Laboratory Asphalt–Aging Protocols

Before undergoing physicochemical and rheological characterization, the fresh straight-run AP-5 asphalt along with its specimens mingled with different percentages of waste shoe polish (e.g., 5, 10 and 15 wt. % WSP) were exposed to accelerated artificial weathering through the rolling thin film oven (RTFO) and/or pressure aging vessel (PAV).

In accordance with ASTM D8272-19 [41], the short-term RTFO aging was conducted by filling eight glass cylinders with roughly 35 ± 0.50 g of fresh fluid bitumen and introducing them in a rolling oven (Model CS325, James Cox & Sons, Inc., Colfax, CA, USA) fed with a stream of hot air flowing at a rate of 4000 mL min^–1^ for 1 h and 25 min at 163 ± 0.50 °C. This laboratory method procedure was especially executed to predict the oxidative aging of the asphalt binder that can occur during the different stages of manufacturing/storage, transportation/placement, or compaction of bituminous mixes.

In accordance with the ASTM D6521-13 test method [42], the long-term PAV aging was performed for 20 h at 100 °C and under 2.10 MPa of air pressure by pouring approximately 50 ± 0.50 g of RTFO-aged asphalt into stainless steel pans and placing them into an air-pressurized PAV (PAV3, Applied Test Systems LLC, Butler, PA, USA). This technique was particularly applied to forecast in-service aging that could happen within 5~10 years.

The air bubbles produced during the PAV aging were removed by degassing the binder by means of a VDO 81-PV2610 (Vacuum Degassing Oven, NOVA Measurements LLC, Atlixco, Pueblo, México) at 170 °C for half an hour. After aging, all of the asphaltic samples were kept in metal containers that were hermetically sealed and ready for testing right away.

### 2.3. Thin-Layer Chromatography with Flame Ionization Detection (TLC-FID)

The direct effects of several dosages of waste shoe polish (e.g., 5, 10 and 15 wt. % WSP) on the chemical composition of AP-5 asphalt cement prior to and after aging were investigated in depth by using a metallic rack of silica rods (Type Chromarod-S5, LSI Medience Corporation, chiyoda-ku, Tokyo, Japan) mounted on an IATROSCAN^®^ MK6s FID (Iatron Laboratories Incorporation, Tokyo, Japan).

Before proceeding with spotting, the 10-set chromarods (pore diameter of 60 Å, particle size of 5 µm, and length of 15.2 cm) were thoroughly cleaned and activated through the blank scan with the help of hydrogen flame. The flame was fed by an air velocity of 2000 mL min^–1^ and a pure grade hydrogen (H_2_) of 160 mL min^–1^ [22].

An asphaltic sample solution with a concentration of 2% (*w/v*, weight by volume) was prepared by dissolving the binder (or the WSP) with dichloromethane. With the aid of a 5 µL Drummond microdispenser (Drummond Scientific, Broomall, PA, USA), approximately 1 µL of sample solution was carefully spotted on the baseline (origin) of chromarods. The separation of binder into saturates, aromatics, and resins was achieved by partially and successively plunging the chromarod rack into three consecutive closed/developing tanks containing several organic solvents, namely, *n*-hexane for 45 min, toluene for 15 min, and dichloromethane/methanol for 5 min [22]. The asphaltenes remained intact at the starting point.

When the separated SARA fractions, which are linearly spread on the silica chromarod, were burnt with the hydrogen flame, carbon ions were generated and gathered by the collector electrode. The current was intensified along with the signals of carbon ions, which were recorded afterwards. The concentration of saturates, aromatics, resins, and asphaltenes (SARA wt. %) was determined by integrating the four-band zones of each silica gel bar at the base line before and after each band.

After each solvent’s development, the chromarod holder was introduced into a dry chamber for 2 minutes at 85 °C with the purpose of eliminating the solvent residues. For each asphaltic sample, five parallel quartz bars were utilized at a scanning speed of 30 s scan^–1^. For the sake of repeatability and reproducibility, the TLC-FID analysis was carried out 5 times [22].

### 2.4. Fourier-Transform Infrared Spectroscopy (FT-IR)

In an attempt to gain a deeper insight into the effect of numerous doses of waste shoe polish (e.g., 5, 10 and 15 wt. % WSP) on the molecular structure and the chemical composition of unaged and aged base AP-5 asphalt cement, FT-IR spectroscopy was carried out accordingly.

The FT-IR spectra were recorded by means of Hyperion (3000 FT-IR) Spectrometer (Bruker Optics, Ettlinger, Germany) by adopting the following conditions: spectral resolution of 1 cm^–1^, test range of 4000~650 cm^–1^, and 30 sans per sample. By mixing KBr either with the WSP or with the binder, the chemical characterization of discarded shoe polish, base asphalt, and WSP-dosed asphalt specimens was successfully achieved.

Since the carbonyl (C=O) band at 1700 cm^–1^ and the sulfoxide (S=O) band at 1030 cm^–1^ are both susceptible to oxidation, they were employed in tandem to monitor the degree of aging and/or modification using their carbonyl index (CI) and sulfoxide index (SI), which were calculated using the Equations (1) and (2) [43]:

(1) $$Carbonyl\;Index\;\left(\mathrm{CI}\right)=\frac{A_{1700}}{\sum A}$$

(2) $$Sulfoxide\;Index\;\left(\mathrm{SI}\right)=\frac{A_{1030}}{\sum A}$$

where ∑A = A_(2953,2862)_ + A_1700_ +A_1600_ + A_1460_ + A_1376_ + A_1030_ + A_864_ + A_814_ + A_743_ + A_724_.

**Figure 3:**
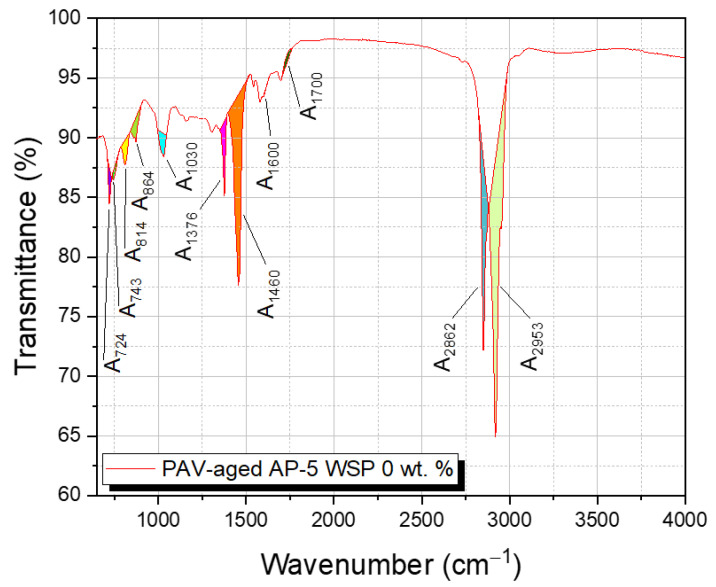
FT-IR spectrograph of PAV-aged plain AP-5 bitumen used as an illustrative example to define the several peak areas with their integration limits.

As exemplified by Figure 3, the aging indices, CI and SI, were obtained by dividing the carbonyl area (A_1700_) and sulfoxide area (A_1030_) by the sum area of peaks (∑A), respectively [43]. The vertical limit peaks with their respective functional groups are summarized in Table 3 [43].

### 2.5. Conventional Binder Tests (Softening Point, Penetration, Viscosity, and Ductility)

In order to obtain further details about the influence of various contents of WSP (e.g., 5, 10 and 15 wt. %) on the physical properties of AP-5 asphalt cement before and after aging, several laboratory techniques were arranged and performed in quadruplicate, namely: penetration, softening point, viscosity, and ductility.

The penetration test, or the degree of softness/hardness of AP-5 bitumen, was measured by employing a Humboldt Mfg Electric Penetrometer (Humboldt Mfg. Co., Elgin, IL, USA) according to the protocol described in ASTM D5 [44]. By allowing a standard 100 g loaded needle to vertically pierce the surface of an asphalt sample for 5 s at 25 °C, the penetration was determined in tenths of a millimeter (mm).

Based on ASTM D36 [45] and with the assistance of the ring-and-ball test apparatus RKA 5 (Anton Paar GmbH, Ashland, Virginia, VA, USA), the softening points of asphalt samples were collected. This test method was not only executed with the intention of determining the bitumen’s consistency level but also of inquiring about the temperature at which the binder would attain a specific viscosity degree (i.e., equiviscous temperature). In short, by placing a steel ball with a diameter of 1 cm on the top of a thin bituminous disk, the consistency was determined by letting the binder flow down in a heat-conditioned distilled water bath for a distance of 2.50 cm.

As directed in ASTM D4402 [46], the rotational viscosity (RV), or more precisely, the internal friction of the binder, was measured by using a Brookfield DV III Rheometer (Brookfield, Middleboro, MA, USA). In this procedure, 10 ± 5.00 g of molten bitumen was poured into a sample chamber, which was then inserted into a thermo-container and maintained at 135 °C (275 °F). The RV was estimated by sensing the torque needed to rotate a SC4-27 spindle at 20 rpm fully immersed in the hot binder.

On the other hand, the elasticity and adhesiveness of asphalt specimens were assessed in accordance with the method detailed in ASTM D113 [47] and by using a ductilometer apparatus (Woojin Precision Co., Ltd., Gwangju-si, Gyeonggi-do, South Korea). Concisely, the ductility (cm) was determined by elongating a standard asphaltic briquette in a water bath kept at 25 °C at a rate of pull of 5 cm min^–1^ until the thread broke (i.e., failure).

### 2.6. Flash Point (FLP) and Fire Point (FIP) Tests

The flash point (FLP) and fire point (FIP) tests were especially carried out to figure out the temperature to which the fresh neat base AP-5 asphalt along with its samples containing various doses of waste shoe polish (e.g., 5, 10 and 15 wt. % WSP) can be heated and handled or processed safely.

The FLP test is described as the lowest temperature where the binder vapors can catch fire in a flash form. In other words, it is the maximum temperature to which asphalt can be heated safely without the risk of an instantaneous flash or spark in the presence of an open flame. Meanwhile, the FIP is also expressed as the lowest temperature where the binder can ignite and burn for at least 5 s due to the presence of a flame source [48].

The FLP and FIP trials were done by heating an 80 g asphaltic sample above its softening point of 48.40 °C at a rate of 5 °C min^–1^ and stirring it at a rotation speed of 60 rpm in a Cleveland open cup [48]. The critical temperatures were determined when a tiny flame travelling over the surface would provoke the volatile hydrocarbon fumes from the binder to momentarily flash (FLP) or ignite and burn for at least 5 s (FIP).

For combustible asphalt cement, the typical value of FLP ranges from 204 to 288 °C [49]. As a result of the fact that the cutback asphalts are often employed at temperatures over their flash points, all of these materials may pose some risk in use and must be handled with caution. The greater the volatility of the solvent in the fluid asphalt, the more dangerous its usage is [49].

### 2.7. Dynamic Shear Rheometer (DSR) Test

By employing a dynamic shear rheometer (DSR) from ThermoFisher (Thermo Scientific^TM^ HAAKE^TM^ MARS^TM^ Rheometer, Thermo Fisher-Scientific, Newington, New Hampshire, USA), the impact of various dosages of waste shoe polish (e.g., 5, 10 and 15 wt. % WSP) on the viscoelastic balance of base AP-5 bitumen was meticulously investigated.

The fatigue cracking was assessed at intermediate temperatures (e.g., 4~40 °C); meanwhile, the permanent deformation (i.e., rutting) was monitored at higher temperatures (i.e., 46~82 °C), following the procedure detailed in ASTM D7175 [50]. The loading frequency of 10 rad s^–1^ (1.59 Hz), which was selected to simulate the shearing action matching a traffic speed of ca. 55 mph (90 km h^–1^), was utilized to issue the several rheological parameters of unmodified and WSP-modified asphalt specimens.

The predictors of long-term serviceability of road pavement—viz. the rutting factor (G*/sin δ) and the fatigue cracking factor (G*.sin δ)—were calculated with the aid of the potential plastic deformation, known as the phase angle (δ), and the stiffness, designated as the complex shear modulus |G*|.

On the basis of scanning temperatures, two bituminous geometries were prepared and tested: for higher temperatures (46~82 °C), 25-mm diameter/1-mm thickness; and for intermediate temperatures (i.e., 4~40 °C), 8-mm diameter/2-mm thickness.

### 2.8. Multiple Stress Creep Recovery (MSCR) Test

In accordance with AASHTO T 350-19 [51], the MSCR test was carried out with the assistance of a dynamic shear rheometer (DSR) manufactured by ThermoFisher (Thermo Scientific^TM^ HAAKE^TM^ MARS^TM^ Rheometer, ThermoFisher Scientific, Newington, New Hampshire, NH, USA). It was conducted with the aim of probing the straight impact of various portions of waste shoe polish (e.g., 5, 10 and 15 wt. % WSP) on the potentiality of base AP-5 bitumen towards rutting, as well as assessing the effectiveness and feasibility of WSP modification.

At a temperature of 64 °C, which symbolizes the average 7-day maximum pavement design temperature in South Korea, a disk-shaped asphaltic specimen with a diameter of 25 mm and a height of 1 mm was inserted between two metallic plates and the MSCR test was run accordingly. The asphalt samples were subjected to ten cycles of loading and recovery at different stress levels, 0.1 and 3.2 kPa, respectively. Only six minutes were required to complete the entire exam. After loading for 1 s, the RTFO-aged bituminous samples were allowed to recover for 9 s.

Certain relevant and accessible metrics were collected at the end of the experiment, such as the percent recovery (*R*%) along with the non-recoverable creep compliance (*J_nr_*), also termed as the rutting potential index. With regard to the overall stress exerted, the *J_nr_* designates the amount of residual stress remaining in the asphaltaneous specimen after undergoing multiple creep–recovery cycles.

The *J_nr_* was utilized to determine how sensitive the binder is to changes in deformation and stress. Meanwhile, the *R*% was employed to see how well the sample reverts back to its initial shape when the load has been lifted. The percent recovery was exploited to ascertain whether or not the binder exhibits elastic behavior or a stress dependency. Using the formulae below, the *J_nr_*_0.1_, *J_nr_*_3.2_, ∆*J_nr_*, and *R%* were calculated:

(3) $$J_{nr\;0.1}\left(\frac{1}{\mathrm{kPa}}\right)=\frac{1}{10}\left\{\sum_{n=1}^{\;10}\left(\frac{\mathrm{Non}-\mathrm{Recoverable}\;\mathrm{Strain}}{0.1}\right)\right\}$$

(4) $$J_{nr\;3.2}\left(\frac{1}{\mathrm{kPa}}\right)=\frac{1}{10}\left\{\sum_{n=1}^{\;10}\left(\frac{\mathrm{Non}-\mathrm{Recoverable}\;\mathrm{Strain}}{3.2}\right)\right\}$$

(5) $$\Delta \;J_{nr\;}\left(\%\right)=\left\{\left(\frac{J_{nr\;3.2}-J_{nr\;0.1}}{J_{nr\;0.1}}\right)\times 100\right\}\;\leq 75\;\%$$

(6) $$R\;\left(\%\right)=\frac{1}{10}\left\{\sum_{n=1}^{\;10}\left(\frac{\mathrm{Recoverable}\;\mathrm{Strain}}{\mathrm{Peak}\;\mathrm{Strain}}\right)\times 100\right\}$$

### 2.9. Bending Beam Rheometer (BBR) Test

The Bending Beam Rheometer (BBR) test was particularly executed to closely investigate the effect of different contents of waste shoe polish (e.g., 5, 10 and 15 wt. % WSP) on the cracking potential of base AP-5 asphalt and its low-temperature stiffness.

The BBR apparatus (ATS Bending Beam Rheometer (BBR2S), Applied Test Systems, ATS, Butler, PA, USA) was operated following the procedure described in AASHTO T313 [52]. Basically, the test was run at two low temperatures of –12 and –6 °C to measure the stiffness values as well as the creep compliance of RTFO+PAV-conditioned blends, which were presoaked in a cold ethanol bath for 1 hour at each specific temperature. By applying a constant load or static stress (100 g or 980 ± 50 mN) at the midspan of each prismatic bituminous beam (geometry: width = 12.70 ± 0.25 mm, thickness = 6.35 mm ± 0.25 mm, length = 127 mm) for several loading times varying from 8 to 240 s, certain specific rates of deformation were extracted as an average result of three identical beam specimens.

The flexural creep stiffness S (t) expressed the resistance of the PAV-binder residue to creep loading, whereas the creep rate “m-value” denoted the fluctuation in binder stiffness (i.e., relaxation rate, m). The creep rate and creep stiffness parameters were obtained after 60 s of loading, which mimics 2 h of loading at 10 °C (18 °F)-chiller temperature.

## 3. Results and Discussion

### 3.1. Thin-Layer Chromatography-Flame Ionization Detection (TLC-FID)

The TLC-FID (Iatroscan), which is considered a modest, quick, and powerful analytical technique, is not only capable of quantifying but also of separating a broad range of hydrocarbon-based oil materials (e.g., shale oils, heavy petroleum, asphalt, and pitch and the like) into four principal categories of constituents. These are typically called SARA generic fractions, which stand for Saturates, Aromatics, Resins, and Asphaltenes, as per their polarity and polarizability, without prior flocculation of asphaltenes [53].

Under unaged and aged conditions, the TLC-FID technique was employed aimed at monitoring the response of SARA fractions of fresh neat base AP-5 asphalt towards the gradual inclusion of waste shoe polish (WSP).

**Figure 4:**
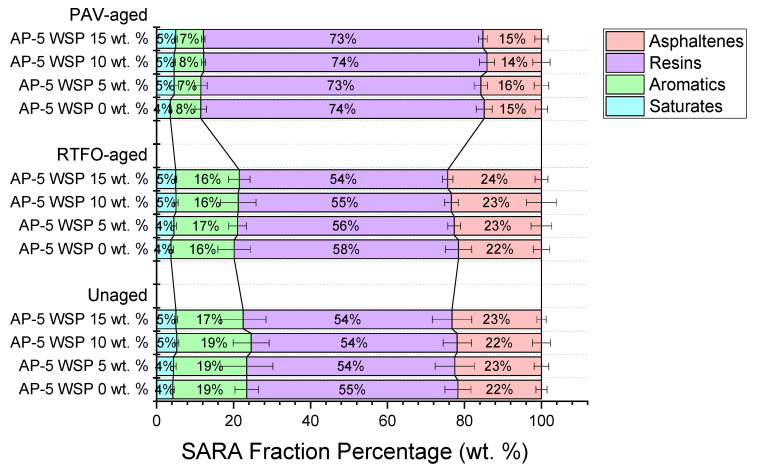
Impact of various dosages of waste shoe polish (e.g., 5, 10 and 15 wt. % WSP) on the SARA generic fractions of base AP-5 asphalt before and after RTFO and PAV aging.

A cursory visual inspection of Figure 4 quickly indicates that the straight-run AP-5 bitumen comprises a large amount of resins (54.87 ± 3.38 wt. %), moderate concentrations of asphaltenes (21.70 ± 1.44 wt. %) and aromatics (19.20 ± 3.03 wt. %), with minor quantities of saturates (4.20 ± 0.50 wt. %). On the other hand, the waste shoe polish (WSP), which was peculiarly impoverished in naphthene aromatics (00.00 ± 00.00 wt. %), was discovered to contain plentiful amounts of asphaltene-like components (e.g., calcium stearate, C.I. solvent yellow 56 (C.I. 11021), and C.I. solvent black 7 (C.I. 50415:1)) with a mass percentage of roughly (56.54 ± 0.67 wt. %). The remaining portions such as saturated hydrocarbons (e.g., hydrotreated light alkanes and paraffin wax) and resinous material (e.g., oleic acid, Carnauba wax, Montan acid wax, glycol montanate, polyethylene and dimethicone) were present in humble doses, 31.58 ± 1.27 wt. % and 11.88 ± 1.76 wt. %, respectively.

By carefully inspecting the data projected in Figure 4, it can be noticed that the continuous feeding of the fresh neat bitumen (i.e., unaged AP-5 WSP 0 wt. %) with the organic additive did not drastically affect either its maltenes or its asphaltene constituents. This could be because the WSP contains a high concentration of volatile diluents (e.g., hydrotreated light alkanes as petroleum solvent), which dissolved the bitumen and then evaporated at different rates depending on their boiling points (≤180 °C), leaving behind a certain quantity of binder that is close to the original one (i.e., similar to cutback asphalt). Notwithstanding, it should be underlined that the cumulative WSP incorporation with its solvents/wax and dyes/pigments resulted in a trivial increase in the blend’s saturates and asphaltenes, respectively.

Furthermore, a detailed examination of the data condensed in Figure 4 clearly reveals that the heating conditioning provoked a gradual growth in the chemical polarity of WSP-loaded bitumen samples in comparison to the raw blend specimens. The laboratory aging protocols had seemingly an obvious influence on the partition of SARA fractions and thus on the chemical structure of asphalt, leading to the generation of more polar components than non-polar ones. In particular, the RTFO- and/or PAV-aged bitumen residues, including various portions of waste shoe polish (i.e., 5, 10 and 15 wt. % WSP), exhibited virtually comparable trends towards accentuated polarity with aging. Owing to their inertness to oxidation, the saturated components were minimally altered after thermal incubation. Nevertheless, as more oxygen was integrated into the aromatic molecules, their contents were reduced and additional resins and asphaltenes were produced. Accordingly, the binder would display lower thermodynamic stability associated with higher viscosity and consistency [54].

The striking divergence observed in asphaltene content was the sole apparent distinguishing trait. The asphaltenes reported a slight rise in the count of aromatics and resins after short-term aging (RTFO) [55]. Nonetheless, they showed an unusually precipitous drop immediately after long-term aging (PAV). This abnormal response of these high-density hydrocarbons against the heat–pressure exposure could chiefly be attributed to the large volume of resins, which were obviously outfitted with a potential asset to percolate throughout the asphaltene micellar cores and relatively fragment them into several resin–asphaltene particles that eventually wound up in the solvent medium [56].

### 3.2. Fourier-Transform Infrared Spectroscopy (FT-IR)

In order to obtain deeper insights into the effect of varying doses of waste shoe polish (e.g., 5, 10 and 15 wt. % WSP) either on its structure or its chemical composition prior to or after aging, the virgin AP-5 bitumen was spectroscopically characterized in detail by means of FT-IR.

**Figure 5:**
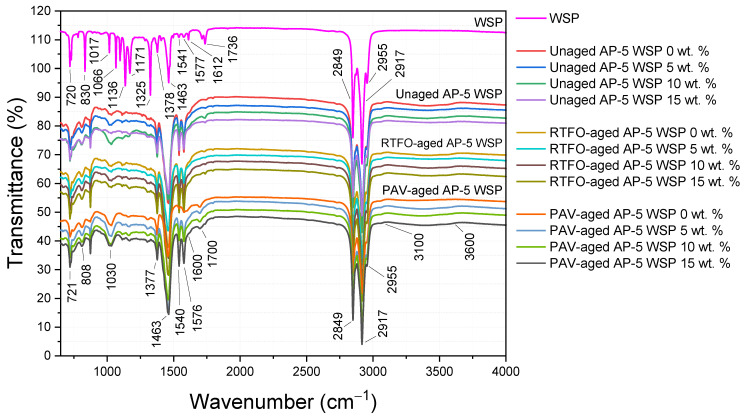
FT-IR spectra of waste shoe polish (WSP), unloaded and base AP-5 bitumen loaded with different WSP portions (e.g., 5, 10 and 15 wt. %) before and after RTFO and PAV aging.

Figure 5 illustrates a bunch of infrared spectra for waste shoe polish (WSP), raw (i.e., unaged), RTFO-, and PAV-aged asphalt specimens. As seen, the spectrum of fresh plain binder (i.e., unaged AP-5 WSP 0 wt. %) gave rise to a very weak broad band located at 3100~3600 cm^–1^, which more probably stemmed from the (O–H) stretching vibrations and/or the (N–H) bending vibrations. The two significant hydrocarbon absorption bands at 2849 and 2917 cm^–1^ are ascribed to the symmetric and asymmetric stretching vibrations of alkyl groups –(CH_2_)_n_, respectively. On the other hand, the C–H asymmetric bending vibrations in the methylene –(CH_2_)_n_ and methyl (CH_3_) groups caused the two peaks at 1463 cm^–1^ and 1377 cm^–1^, respectively. The band corresponding to *in*-plane bending of –CH_2_ was observed at 721 cm^–1^. When these former peaks, which are mostly made up of alkyl groups, are inspected in great detail, it makes sense to assume that the saturates fraction did not significantly react when it was heated or treated with WSP, which proves that they are chemically inert.

Both the bituminous specimens and the discarded shoe polish exhibited the salient feature of aromatic compounds as a weak band near 1600 cm^–1^. This band occurred as a result of the C–C stretching vibrations in arenes. At lower stretching frequencies, a series of small peaks, viz., 850, 723, and 548 cm^–1^ (data not shown), took place, and they mainly originated from the C–H wag vibrations of para-, meta-, and ortho-isomers in benzene rings. All the bands connected to aromaticity were evidently altered by the combined effects of aging and additive treatment and showed a steady increase in signal intensity, revealing an incessant growth in the yield of resins and asphaltenes, the heaviest fractions of bitumen.

The WSP spectrum revealed a single peak at about 1171 cm^–1^, which is presumably related to the C–O stretching vibration and is typically overlaid within the 1300~1000 cm^–1^ region. The existence of carbon–oxygen double bonds, often known as carbonyl groups (C=O stretching), can be easily discerned by the signal at 1736 cm^–1^ in the spectrograph [57]. These findings vindicate the presence of ester groups in the SP (i.e., shoe polish) basic formula, which are mostly derived from oleic acid, Montan acid wax, Carnauba wax, and glycol montanate, etc. Moreover, the organic additive exhibited the characteristic features of calcium stearate (i.e., C_36_H_70_CaO_4_) at 1541 and 1577 cm^–1^, which were induced by the symmetric and asymmetric stretching vibrations of carboxylate ions (R–COO^–^) bound with calcium ions (Ca^2+^). The alkyl chains in the C_36_H_70_CaO_4_ molecular structure created several peaks at 1463, 2849, and 2917 cm^–1^, which came from methylene scissoring, and symmetric and antisymmetric methylene stretching vibrations (i.e., δ_s_CH_2_, ν_s_CH_2_, and ν_a_CH_2_), respectively [58].

The process of oxidation involves the integration of oxygen into the asphalt’s molecular structure. When asphalt oxidizes, two major byproducts are created: (a) carbon (C) is oxidized to carbonyl (C=O); and (b) sulfur atoms (S) are oxidized to sulfoxide (S=O) [59].

**Figure 6:**
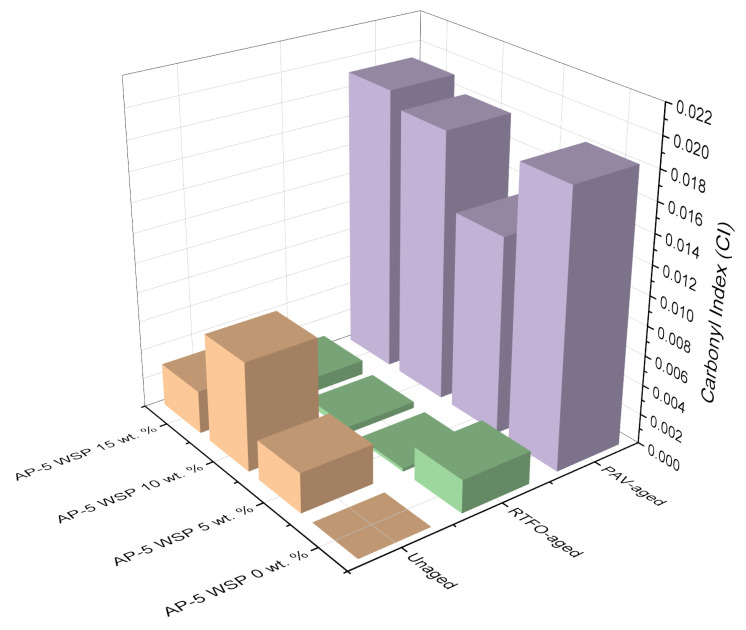
Effect of various WSP dosages (e.g., 5, 10 and 15 wt. %) on the carbonyl index (CI) of base AP-5 asphalt before and after RTFO and PAV aging.

**Figure 7:**
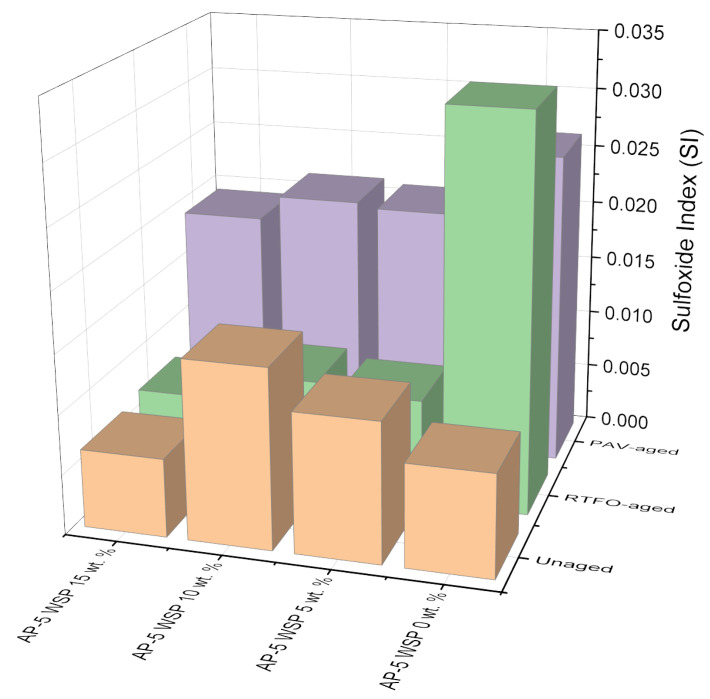
Effect of various WSP dosages (e.g., 5, 10 and 15 wt. %) on the sulfoxide index (SI) of base AP-5 asphalt before and after RTFO and PAV aging.

Referring to Figure 5, Figure 6 and Figure 7, it can be perceived that the sulfoxide index (SI) of unmodified asphalt (i.e., unaged AP-5 WSP 0 wt. %) increased sharply immediately after RTFO-exposure, as did the intensity of the broad band (S=O) at 1030 cm^–1^. Meanwhile, the carbonyl index (CI), along with the intensity of the (C=O) band at 1700 cm^–1^, which was absent in the unaged virgin binder but present in the WSP, also increased after PAV incubation. The (S=O) band reflects the short-term aging that happens during manufacturing, transport, and compacting processes, whereas the (C=O) band mirrors the long-term aging that occurs during pavement service [60].

Aging had a stronger impact on sulfoxide components than on carbonyl ones, as evidenced by the consistent rise in their aging indices and the constant sharpening and widening of their FT-IR signals, suggesting their higher susceptibility to oxidation. This might be attributed to the fact that sulfur in bitumen is more reactive towards oxygen than carbon, hence causing sulfoxides to emerge earlier than carbonyls [61].

In contrast to the 15 wt. % dosage, the changes produced by the addition of 5 and 10 wt. % WSP in the infrared spectra of unaged asphalt mixtures showed that the oleic acid, Carnauba wax, Montan acid wax, glycol montanate, calcium stearate, and hydrocarbon waxes (petroleum)–clay-treated microcrystalline, among others, reinforced the band absorbances of carbonyl at 1700 cm^–1^ and sulfoxide at 1030 cm^–1^.

At this stage, it is reasonable to assume that when the ingredients of discarded shoe polish (i.e., mainly resinous ones) were added at lower and intermediate dosages (i.e., 5 and 10 wt. % WSP), they did not sufficiently peptize the asphalt constituents (i.e., particularly asphaltenes) and instead remained flowing in the dispersion medium with their carbonyl and sulfoxide compounds, thereby contributing to the continuous growth of oxidation peaks in the FT-IR spectrograph [62]. Nevertheless, upon their introduction at a larger dosage (i.e., 15 wt. % WSP), the rejuvenation effect started to take place by peptizing the numerous asphaltene micelles with more resin, hence, resulting in a noticeable attenuation of the oxidation peaks [62].

Interestingly, the prolonged treatment of base AP-5 bitumen with the additive caused the CI and SI to decrease throughout both the short-term and long-term aging, revealing the lubrication and anti-aging potential of discarded shoe polish. Oleic acid (18:1), the predominant unsaturated fatty acid of olive oil, is mostly responsible for the acquisition of these qualities [63,64]. When added to bitumen, it could potentially suppress the oxidation through its rejuvenating action [63,64].

The outcomes of oxidation products, viz., sulfoxides and carbonyls, are straightly associated with a remarkable increment in larger molecular-size polar constituents, strikingly asphaltenes and resins, which govern the whole viscoelastic solid behavior of asphalt binder. Increasing the stiffness would increase the likelihood of road pavement cracking [65].

As evidenced by Figure 5, there were no new/extra peaks developed following the incremental treatment of AP-5 binder with WSP, justifying the fact that no major chemical changes occurred and no new compounds were produced.

Lastly, it is critical to emphasize that relying solely on FT-IR qualitative and quantitative analysis to predict the aging and/or modification performance of asphalt cement is not always trustworthy, especially when certain misleading conclusions could be drawn [66]; accordingly, further detailed studies are needed.

### 3.3. Conventional Binder Tests (Penetration, Softening Point, Viscosity, and Ductility)

A wide array of laboratory tests was performed on the raw (i.e., unaged), RTFO-, and PAV-aged binders containing several fractions of waste shoe polish (e.g., 5, 10 and 15 wt. % WSP), aimed at investigating the combined impacts of additive and thermal conditioning on their physical properties. Tests included penetration, softening point, viscosity, and ductility.

**Figure 8:**
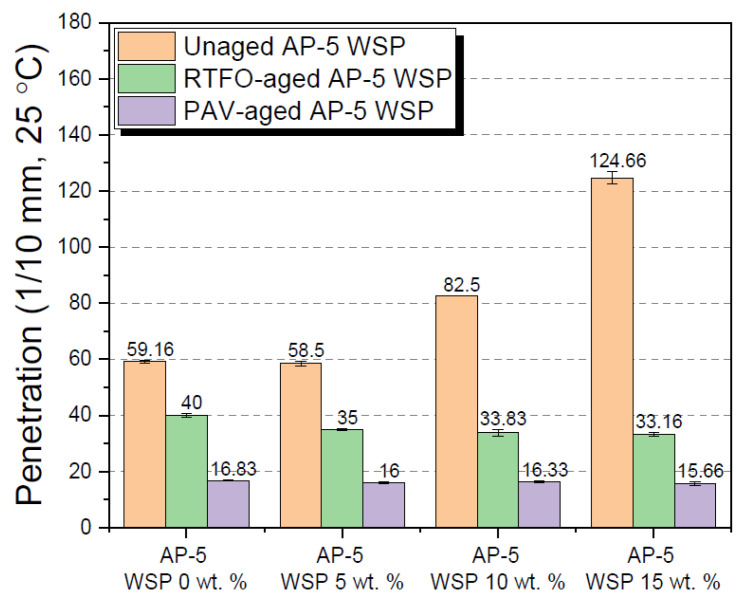
Influence of various WSP concentrations (e.g., 5, 10 and 15 wt. %) on the needle penetration of base AP-5 asphalt before and after RTFO and PAV aging.

**Figure 9:**
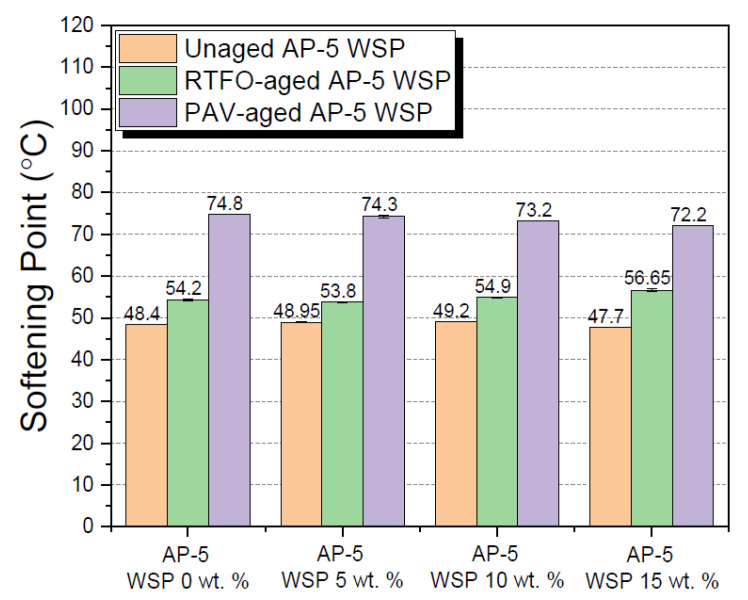
Influence of various WSP concentrations (e.g., 5, 10 and 15 wt. %) on the ring-and-ball softening point of base AP-5 asphalt before and after RTFO and PAV aging.

**Figure 10:**
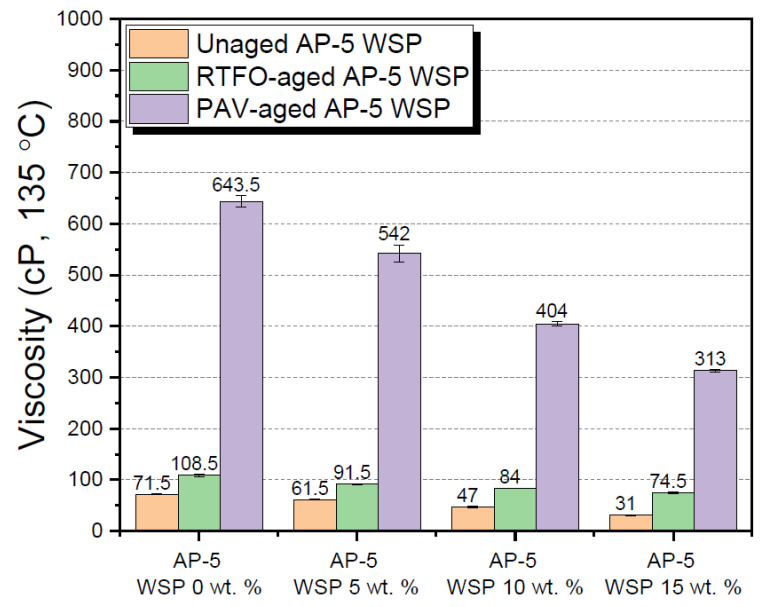
Influence of various WSP concentrations (e.g., 5, 10 and 15 wt. %) on the viscosity of base AP-5 asphalt before and after RTFO and PAV aging.

**Figure 11:**
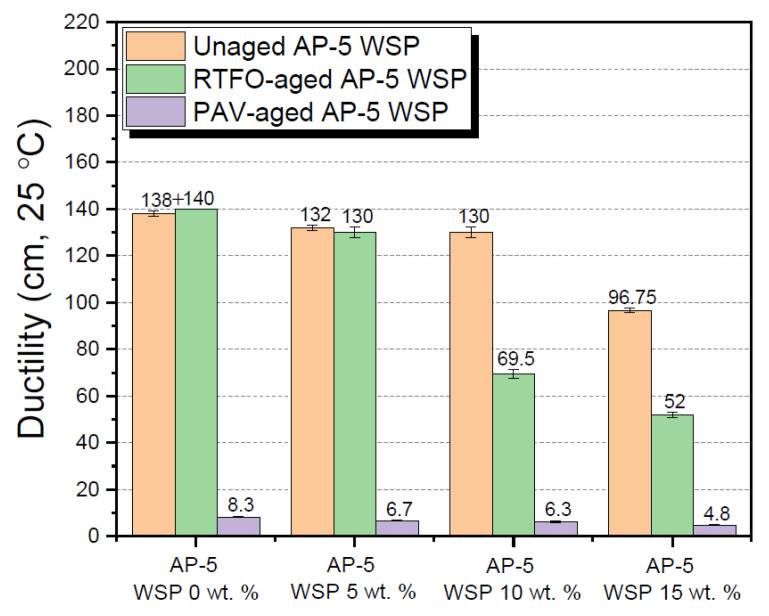
Influence of various WSP concentrations (e.g., 5, 10 and 15 wt. %) on the ductility of base AP-5 asphalt before and after RTFO and PAV aging.

Figure 8, Figure 9, Figure 10 and Figure 11 show the test results for the straight-run (i.e., original) and artificially weathered asphaltic specimens. As seen from Figure 8, the gradual introduction of modifier into the neat base bitumen greatly boosted its penetration number to a certain extent, which is actually caused by the basic WSP ingredients mainly consisting of waxy colloidal emulsion (e.g., solvents, wax, plasticizers, and texturing agents, etc.) that tended to lower the consistency (i.e., hardness) of the fresh bituminous specimens. The application of fluxing agent in conjunction with short- or long-term aging has curiously stiffened the binder. The intrinsic features of WSP such as its ability to dissolve and evaporate may account for the considerable expansion or contraction observed at the penetration level, resulting in a matrix with a lower or higher density than that of the original bitumen. It is noteworthy to mention that there was a strong correlation between changes in penetration outputs and resin percentages as the material aged.

The findings of the ring-and-ball test are graphically recapitulated in Figure 9. As shown, the inclusion of 5 or 10 wt. % WSP into the unaged virgin asphalt slightly improved its thermal softening, whereas the use of 15 wt. % marginally reduced it. In contrast, the binder’s melting point (T_R&B_) was enhanced by the RTFO-aging process and minorly depressed by the PAV-aging process as a result of constant treatment with the modifier. With this data in hand, it can be predicted that a higher dose of light solvent may ease the handling of the asphalt cement during the production of bituminous mixes and fight against the occurrence of unexpected and sudden cracking events in road pavements.

Under unaged and aged conditions, Figure 10 demonstrates that fluxing the base AP-5 asphalt cement with different concentrations of waste shoe polish (WSP) lowered the viscosity value remarkably. The consistent increase in fluidity as well as mobility might be attributed to an obvious increase in volatile diluents (e.g., hydrotreated light alkanes and 1-choloro-4-trifluoromethylbenzene (PCBTF)) within the bulk matrix of the binder. Thanks to this, the asphalt–WSP blends should improve the mix workability at lower temperatures by coating the aggregates/stone particles uniformly and lubricating them generously. Furthermore, the temperature at which the manufacturing, transportation, and compaction of bitumen mixtures are performed will be reduced considerably, resulting in more energy consumption savings while concurrently improving the working environment. However, an extremely low viscosity is not recommended since the adhesive properties of blends will be degraded along with the overall performance of bitumen mixes.

A proper examination of Figure 11 reveals that the cohesive strength or extensibility (cm) of asphalt–diluent combinations decreased as the volume of diluent in the mixtures increased, and this was true for both the unweathered and artificially weathered asphalt specimens. The change in ductility may be caused by the loss of low-molecular weight hydrocarbons (e.g., saturates and aromatics) and the increase of polar components in asphalt mixtures (i.e., mainly asphaltenes).

The findings of both penetration and viscosity concur very well with those reported by Wami and co-workers. To create a cutback asphalt for priming road surfaces, these researchers mixed one low-penetration-grade bitumen (45 Pen) with three lighter petroleum products, namely, kerosene, diesel, and lubricating oil, and discovered that as the diluent concentration increased in the blends, the penetration value increased and the viscosity decreased [7].

The data are also in accord with the observations of Shajeev and Koshy [67], which showed that the modification of fresh VG30 bitumen with kerosene at different concentrations of 1, 1.5 and 2 wt. % by binder’s weight caused a magnificent spike in penetrability while reducing the outcomes of viscosity/softening point/ductility.

In an effort to enhance the low-temperature grade, Villanueva and colleagues [68] treated the asphalt with various concentrations of lubricating oil (e.g., 1, 2, 4 and 7 wt. %) and noticed that the penetration value rose considerably while the softening point declined.

The inconsistency found at the softening point level could be strongly attributed to the complex formula of shoe polish, consisting of not only solvents but also waxes, plasticizers, texturizing agents, and dyes. When these latter are added together, they may impact the bitumen softness in various ways, depending on their inherent and newly acquired physicochemical qualities. Hence, this might account for the slight uptick and not downtick in T_R&B_ seen under unaged and short-term aged conditions.

### 3.4. Flash Point (FLP) and Fire Point (FIP) Tests

Under specific testing conditions, the flash point (FLP) of a particular bitumen grade is defined as the lowest temperature at which its combination of volatile vapors–fumes can instantaneously catch fire with air in the form of a flash or spark. The lower the FLP temperature, the greater the binder’s propensity to catch fire. In other words, the higher the FLP temperatures, the safer the binder. On the other hand, the fire point (FIP) refers to the lowest temperature at which the binder traps fire and burns under certain definite circumstances [49].

**Figure 12:**
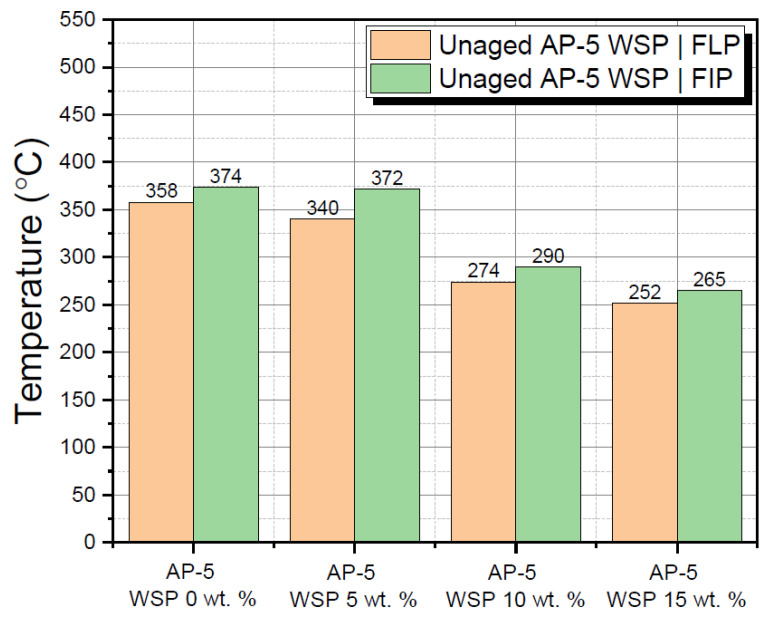
Effect of different WSP contents (e.g., 5, 10 and 15 wt. %) on the flash point (FLP) and fire point (FIP) of unaged base AP-5 asphalt cement.

To ensure their safe handling and processing, the flash point (FLP) and fire point (FIP) tests were conducted on the raw plain base AP-5 bitumen together with its samples fluxed with multiple shots of waste shoe polish (e.g., 5, 10 and 15 wt. % WSP). As evinced by Figure 12, without posing any serious risk or danger to worker health, treating the binder with a gradually increasing concentration of WSP led to a consistent drop in the FLP (T_critical_ > 230 °C) [69] and FIP (T_critical_ > 235 °C) [69] values of asphaltic blends, thereby indicating the existence of potentially highly volatile/flammable/combustible components in their composition, such as hydrotreated light petroleum distillates and 1-chloro-4-trifluoromethylbenzene (PCBTF), among others.

Accordingly, to avoid perilous incidents, when dealing with the hot modified asphalt either during storage and transportation or throughout heating, manufacturing, or compaction stages, the temperature on the field should always be maintained below the critical temperatures measured by FLP and FIP.

These conclusions back up the research of Tadele and Quezon [70], who showed that rejuvenating reclaimed bitumen with numerous fractions of waste engine oil (e.g., 2, 5 and 10 wt. % by weight) resulted in a significant drop in FLP and FIP of asphalt mixtures.

### 3.5. Dynamic Shear Rheometer (DSR) Test

The dynamic shear rheometer (DSR) test was executed to find out how different amounts of waste shoe polish (e.g., 0, 5, 10 and 15 wt. % WSP) affected the propensity of fresh plain base AP-5 asphalt towards rutting and fatigue cracking prior to and after short-term aging (RTFO) and/or long-term aging (PAV).

**Figure 13:**
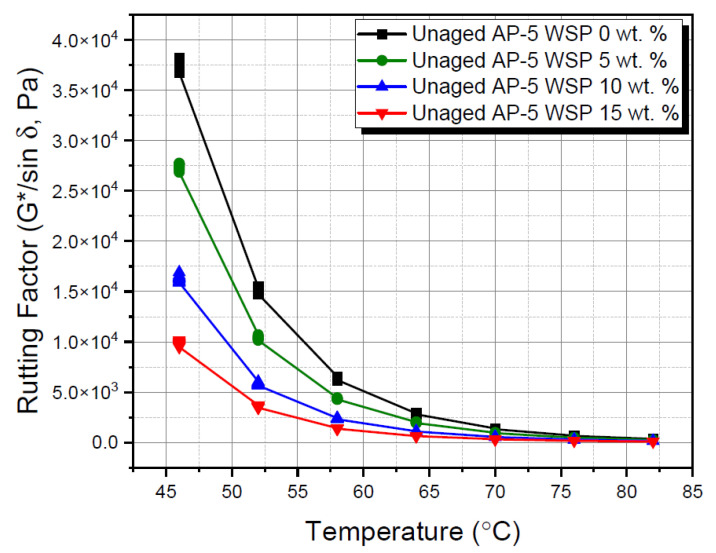
Rutting factor (G*/sin δ) versus temperature for unaged base AP-5 asphalt and its samples containing different WSP percentages (e.g., 5, 10 and 15 wt. %).

**Figure 14:**
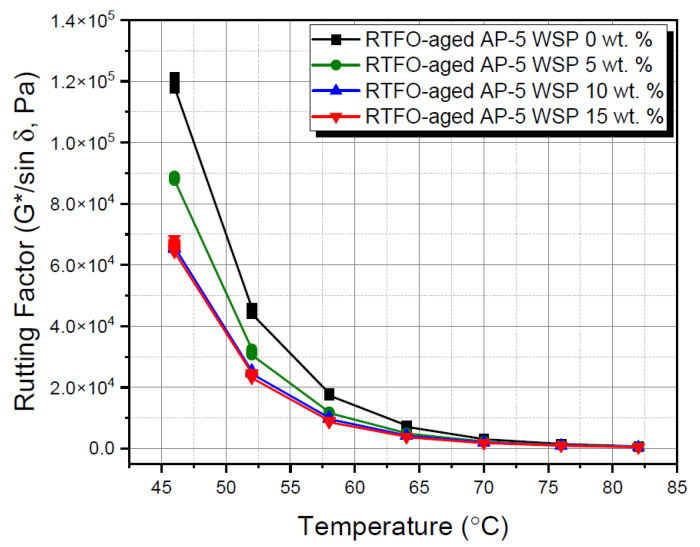
Rutting factor (G*/sin δ) versus temperature for RTFO-aged base AP-5 asphalt and its samples containing different WSP percentages (e.g., 5, 10 and 15 wt. %).

The subjection of WSP-blended asphalt samples either to a regular mix at operating temperature (i.e., 180 °C) or to RTFO aging has revealed dramatic implications, as evidenced by Figure 13 and Figure 14, respectively.

The raw plain bitumen sample (i.e., unaged AP-5 WSP 0 wt. %) was shown to have the lowest potential for permanent deformation (i.e., rutting) when compared to other fresh-blended asphaltic specimens, as indicated by Figure 13. In addition, by increasing both the testing temperatures and the WSP dosage within the asphaltic bulk material, a general tendency towards decline was achieved by the stiffness parameter (G*/sin δ). These findings were found to be in tune with those obtained from conventional test methods, where the unaged modified binders were characterized by lower consistency associated with lower viscosity and stretchability. All the investigated bituminous samples, when tested at temperatures lower than 52 °C, were able to satisfy the essential requirements of the Superpave (i.e., Superior Performing Asphalt Paving) Specifications (i.e., G*/sin δ ≥ 1 kPa) [41]. Specifically, the straight-run AP-5 asphalt along with its samples containing several portions of WSP (e.g., 5, 10 and 15 wt. %) met the requirements satisfactorily at 70, 64, 64 and 58 °C, respectively.

Figure 14 is a schematic representation of the relationship between the rutting factor of RTFO-weathered bituminous specimens and scanning temperatures under the impact of WSP. In the same manner, as the WSP concentration increased with the testing temperatures, the rutting response increased as well. The rich shoe polish (SP) formula, which contains a significant amount of solvents and wax, appeared to be the primary reason for the change in permanent deformation. The short-term weathered binders spiked with multiple shots of additive (e.g., 0, 5, 10 and 15 wt. % WSP) resisted rutting until 70, 70, 64 and 64 °C, respectively (G*/sin δ ≥ 2.2 kPa) [41]. The steady degradation of the rutting index indicates an apparent defect in permanent deformation caused by the additive, and if overused, it cannot confer the bitumen carpet (i.e., road surface) with the necessary strength to endure repeated traffic loading during hot seasons.

**Figure 15:**
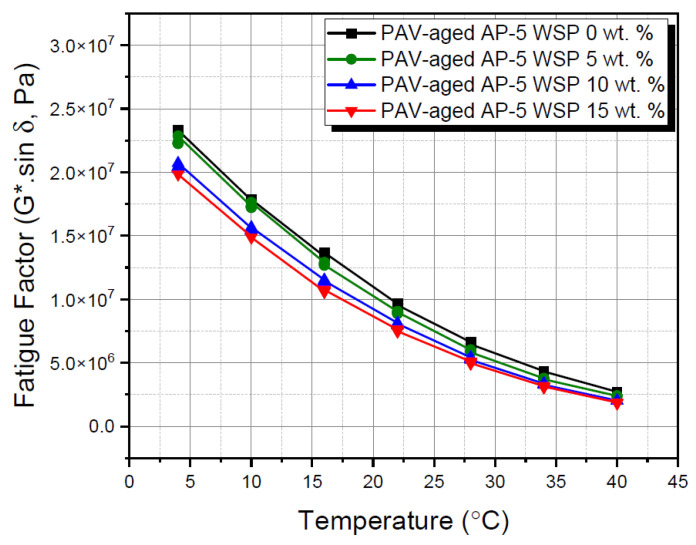
Fatigue cracking factor (G*.sin δ) versus temperature for PAV-aged base AP-5 asphalt and its samples containing different WSP percentages (e.g., 5, 10 and 15 wt. %).

The fatigue cracking factor (G*.sin δ) witnessed a progressive decline tendency following the treatment of base AP-5 asphalt with various WSP amounts, as shown in Figure 15. In terms of shearing energy dissipation, lower (G*.sin δ) values demonstrated that the resins-rich blends were effectively capable to withstand crack propagation. All the RTFO/PAV-incubated binder mixtures complied perfectly with the Superpave regulatory requirements (i.e., G*.sin δ ≤ 5000 kPa) [42] at intermediate temperatures of 34, 34, 34 and 28 °C, regardless of whether they comprised waste shoe polish or not (e.g., 0, 5, 10 and 15 wt. % WSP, respectively). On the whole, it can be forecasted that the usage of a suitable quantity of additive may impart the road surface with sufficient anti-cracking assets, but not with anti-rutting ones if excessively employed.

The current DSR data are in good agreement with prior studies, which have shown that adding softeners (e.g., waste engine oil [71,72], paraffinic oil [73], bio-oil [74], naphthenic recycling agent [75] and waste cooking oil [76], etc.) to the asphalt binder enhances fatigue resistance while compromising the rutting resistance.

### 3.6. Multiple Stress Creep Recovery (MSCR) Test

Since it simulates the actual loading conditions for road surfaces, the multiple stress creep recovery (MSCR) test was manipulated at elevated temperatures with the purpose of accurately examining the elasticity behavior of base AP-5 asphalt and its stress–strength dependencies. To do so, the influence of various fractions of waste shoe polish (e.g., 5, 10 and 15 wt. % WSP) on the rutting performance of short-term aged bitumens was investigated with the aid of the dynamic shear rheometer (DSR) from ThermoFisher.

**Figure 16:**
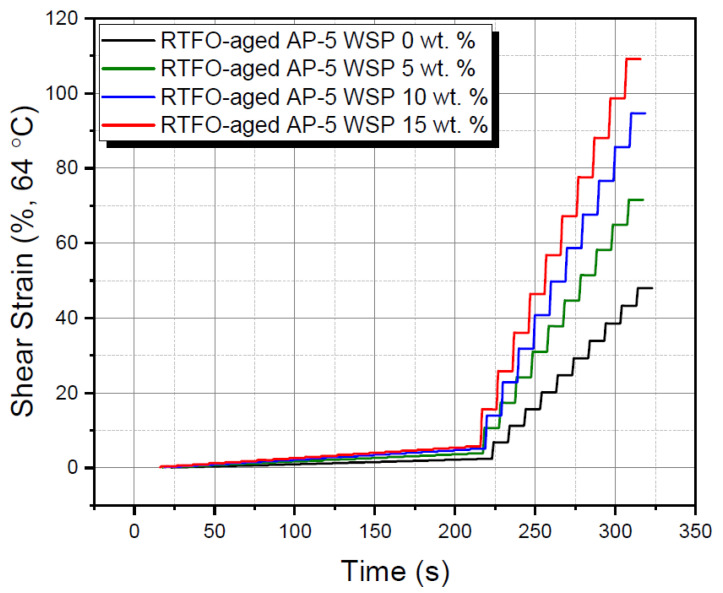
Multiple stress creep recovery (MSCR) test outcomes attained at 0.1 kPa, 3.2 kPa, and 64 °C, for RTFO-aged base AP-5 asphalt and its specimens containing varying WSP percentages (e.g., 5, 10 and 15 wt. %).

Figure 16 shows the MSCR data at 0.1 and 3.2 kPa. The 0.1 kPa labels the behavior of bitumen within the linear viscoelastic zone, whereas the 3.2 kPa describes its behavior beyond the linear viscoelastic zone (i.e., non-linear) [77].

As a rule of thumb, when a given RTFO-aged binder possesses a larger proportion of recoverable strains (*R*%), it has better anti-rutting capacity [78]. In comparison to WSP-modified asphalt specimens, the control bitumen (i.e., RTFO-aged AP-5 WSP 0 wt. %) was identified with the greatest *R*% value, suggesting that the stepwise inclusion of the hydrocarbon solvent into the binder might not improve the bituminous mixes’ resistivity to permanent deformation, and hence their high-temperature performances.

From Table 4, it is pertinent to note that all the admixtures were characterized by greater *J_nr_* (i.e., non-recoverable creep compliance) values, with respect to the virgin binder. Hence, rutting is among the distress failures that might occur with asphaltic samples enclosing higher WSP doses. In the AASHTO M 332 Standard Specification [79], the MSCR test and *J_nr_*_3.2_ were employed together to define in great detail the performance requirements of bitumen against rutting. It is stipulated that *J_nr_*_3.2_ ≤ 0.5 kPa^–1^ for extremely heavy traffic (E) loading, *J_nr_*_3.2_ ≤ 1.0 kPa^–1^ for very heavy traffic (V) loading, *J_nr_*_3.2_ ≤ 2.0 kPa^–1^ for heavy traffic (H) loading, and *J_nr_*_3.2_ ≤ 4.0 kPa^–1^ for standard traffic (S) loading.

Scanning the data synthesized in Table 4, it can be seen that the straight asphalt (i.e., RTFO-aged AP-5 WSP 0 wt. %) could withstand (H) loading, whereas the different blends could tolerate (S) loading, versus rutting at 64 °C. Despite the fact that the Superpave Performance Grading (PG) sets the primary requirement of *J_nr_* at 3.2 kPa, the shear stress of 1.0 kPa is also vitally crucial in the protection of certain overly fragile binders against the change in volume and shear stress. Correspondingly, the AASHTO M 332 [79] imposed another important and restrictive criterion, demanding that the variation in *J_nr_* values between 0.1 and 3.2 kPa must be less than 75%.

As shown in Table 4, all the asphaltic specimens met the requirements satisfactorily, revealing an obvious lower sensitivity towards shear stress oscillation. On the other hand, the mixtures containing 5 and 10 wt. % WSP exhibited smaller ∆ *J_nr_* values, as compared to the plain bitumen. This fact suggests that employing either minor or moderate amounts of additive does not have discernable effects on the *J_nr_* sensitivity towards shear stress fluctuations. In other words, whether subjected to excessively higher temperatures or confronted with unexpectedly massive loads, the blends in question are unlikely to develop significant strains.

Under a standard heightened operating temperature of 64 °C, Figure 16 reports the variation of accumulated strain (%) as a function of time (s). A closer inspection of this figure reveals that the modifier had a noticeable effect on the recovery and creep variables. The continuous feeding of straight binder with the fluxing agent caused the accumulated strains to increase gradually, after undergoing numerous stress–relaxation (i.e., creep) cycles. This implies that there is no doubt that excessive WSP use may have a negative impact on permanent deformation.

The MSCR study produced results which corroborate the findings of a great deal of the previous work in this field [80,81,82].

### 3.7. Bending Beam Rheometer (BBR) Test

The bending beam rheometer (BBR) test was employed to assess the impact of various additions of waste shoe polish (e.g., 5, 10 and 15 wt. % WSP) on the rheological properties of base AP-5 bitumen at lower temperatures.

To guarantee that the binder has excellent anti-cracking performance at cooler temperatures, the Superpave^TM^ (i.e., Superior Performing Asphalt Paving) Specifications necessitate that the stiffness value S (t = 60 s) ≤ 300 MPa and m (t = 60 s) or “m-value” ≥ 0.300, at a specific temperature PG grade [52]. When performance grading a bitumen, the BBR test should be conducted at a temperature 10 °C superior to the low grading temperature. For instance, a PG 70–22 asphalt binder would be examined utilizing the BBR instrument at –12 °C.

**Figure 17:**
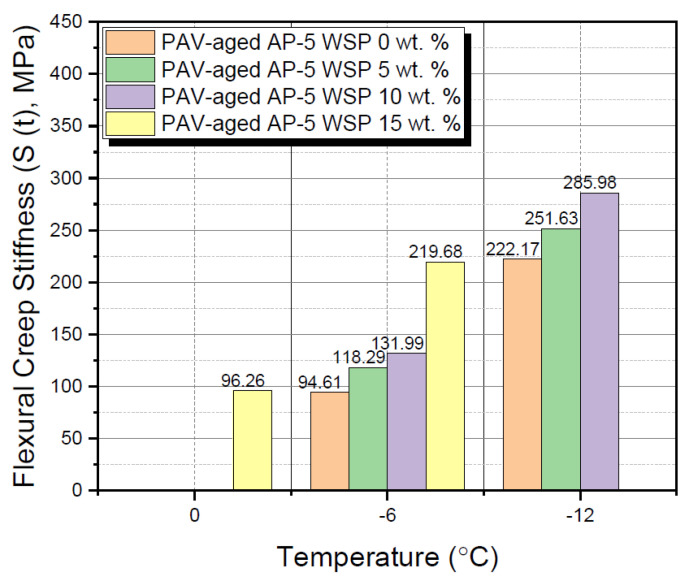
Impact of various WSP fractions (e.g., 5, 10 and 15 wt. %) on the flexural creep stiffness S (t) of PAV-aged base AP-5 asphalt.

**Figure 18:**
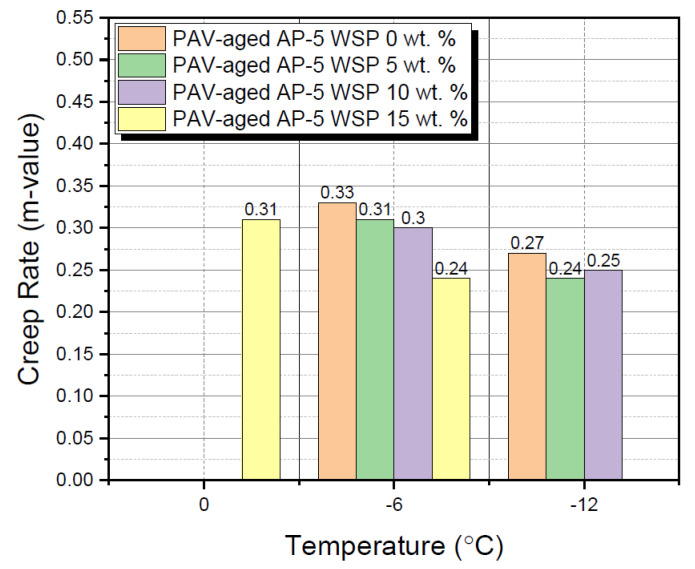
Impact of various WSP fractions (e.g., 5, 10 and 15 wt. %) on the creep rate (m-value) of PAV-aged base AP-5 asphalt.

Figure 17 and Figure 18 illustrate the results of the BBR test carried on the neat and WSP-modified asphalt samples at different temperatures of 0, –6, and –12 °C. When the temperature dropped downwards, it was found that the continuous introduction of fluxing agent into the binder had curiously led to a gradual growth in the creep stiffness modulus S (t = 60 s) associated with a steady decrease in the creep curve slope m (t = 60 s) (m-values), thereby revealing an obvious decline in the low-temperature anti-cracking property. At –6 °C, only the blends containing 0, 5 and 10 wt. % WSP met the specification, as did the asphaltic mixture containing 15 wt. % WSP at 0 °C. This relatively unfavorable outcome could be largely attributed to the blend of wax/fatty acids (e.g., oleic acid, Carnauba wax, Montan acid wax, glycol montanate, paraffin wax, and hydrocarbon waxes (petroleum)–clay-treated microcrystalline) derived from the discarded shoe polish, which has different crystallization degrees or physical hardening effects when added to bitumen at different rates [83].

Lower “m-values” are usually accounted for by a slower relaxation rate, which causes higher stresses that will eventually end up with transverse/thermal cracking. In order to take more advantage of the additive, it is strongly recommended to reasonably apply it in climate zones where the lowest temperature exceeds 0 or –6 °C.

### 3.8. Performance Grade (PG) Test

The data extracted from DSR and BBR were used together with the Superpave performance classification system [69] to evaluate the influence of various quantities of waste shoe polish (e.g., 5, 10 and 15 wt. % WSP) on the performance grade (PG) of base AP-5 asphalt.

**Figure 19:**
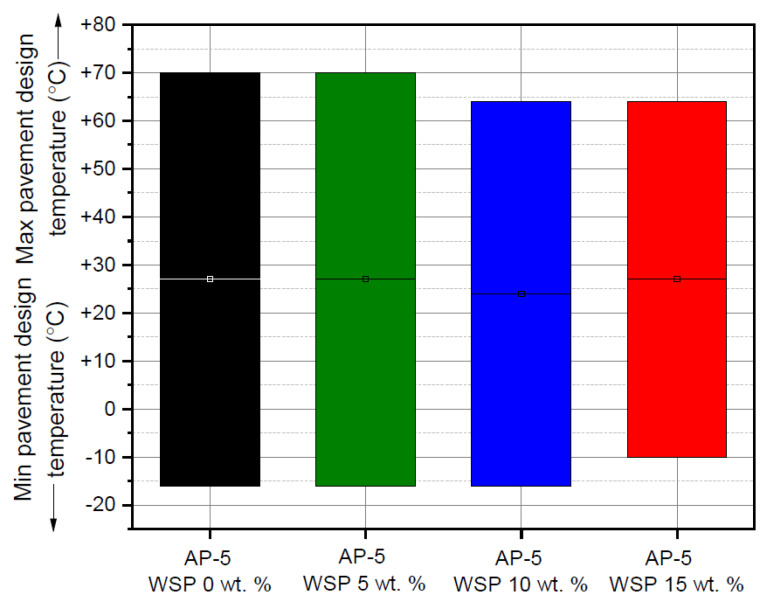
Impact of various WSP doses (e.g., 5, 10 and 15 wt. %) on the performance grade (PG) of base AP-5 asphalt cement.

Figure 19 displays the impact of WSP on the PG of bitumen. The PG 70–22 specifies that the original form of AP-5 bitumen performs satisfactorily under regular traffic loads at temperatures ranging from –22 to +70 °C. In general, the PG system identifies the climatic optimal functioning conditions of certain binders via requirements for both hot- and cold-temperature characteristics of bitumen, which are related to the performance of road paving.

Based on the data plotted in Figure 19, it can be seen that the incremental incorporation of the additive into the binder did not affect either its upper or its lower performance grade at a dosage of 5 wt. %. However, following the addition of 15 wt. % WSP, one grade was lost at lower temperatures, while another grade was lost at higher temperatures after the immediate addition of 10 and 15 wt. % WSP, respectively. In the light of these findings, it can be argued that the modification of the binder with the hydrocarbon solvent has allowed the creation of several performance-graded asphalt binders with a given set of features, which can ultimately be used in a variety of industrial or paving applications (e.g., airports, urban roads and highways, etc.); however, it is strongly advised to work within adequate dose ranges of additive to avoid unexpected adverse events.

## 4. Conclusions

This work was performed with the intention of ascertaining whether or not waste shoe polish (WSP) has the potential to be explored as a fluxing agent for the construction of high-quality paved roads.

To meet this goal, a comprehensive investigation of the influence of several amounts of WSP (e.g., 5, 10 and 15 wt. %) on the performance of base AP-5 asphalt cement was carried out, utilizing a wide range of laboratory techniques.

For instance, the TLC-FID analysis showed that the SARA generic fractions underwent only minor shifts when the binder was treated with WSP and/or artificial aging.

On the other hand, the FT-IR scan established that the progressive administration of WSP into the binder caused no major chemical changes in the combinations, but did improve their anti-aging properties.

Without sacrificing regular ring-and-ball value, the empirical test methods primarily emphasized that the additive has greatly expanded the penetration and reduced the overall binder viscosity, which in turn will lead to a substantial reduction in the manufacturing and application temperatures of asphalt mixtures. Because of this, the need for fossil fuels and energy, as well as concerns about the environment, would drop drastically, and the working conditions would greatly improve.

Even though the continuous loading of binder with the light solvent lessened its flash point (FLP > 230 °C) as well as its fire point (FIP > 235 °C) to some extent, it can still be safely handled without fear of catching fire.

According to the DSR/MSCR/BBR test findings, the additive positively impacted the fatigue cracking property at intermediate temperatures, but negatively altered the high-temperature rutting resistance and low-temperature thermal cracking performance of fluxed bituminous mixtures, eventually generating a wide range of paving grade (PG) bitumina that could be employed in numerous roofing, paving, and industrial applications.

Aside from recycling, incorporating discarded shoe polish into hot-mix asphalt mixes could offer a number of key benefits, including improved pumpability, processability, and compactability of asphalt blends at relatively lower temperatures, as well as safe handling. Along with this, the low VOC-cutter/cutback agent could deliver robust adhesion binder aggregates combined with minimal moisture-induced damage (i.e., stripping) for surface dressings on standard trafficked roadways.

Adding WSP at a rate of less than 10 wt. % of the bitumen’s weight might achieve the optimum balance of engineering properties in the blends. However, the ultimate dosage should be determined on site depending on the application purpose, the composition and characteristics of asphalt concrete mixtures, whether or not the binder is modified, and the ambient environmental conditions (e.g., T °C, pressure, humidity and sunlight, etc.).

Overall, this study has initially proven the effectiveness and feasibility of waste shoe polish (WSP) to be safely employed as a bitumen modifier or partly bio-sourced flux oil to boost the performance and promote the durability of road pavements, while mitigating the pollution posed by the footwear-care market.

To take further green steps, our specialists are currently working on creating some innovative solutions to cope with the several issues encountered in the permanent deformation and thermal cracking properties of asphalt admixtures.

Extensive research efforts are also underway to further assess the direct impact of WSP on the bitumen attributes in terms of compatibility, storage and stability, coating ability and moisture resistance, solubility, hardness, elasticity, and so on. This will definitely enable the new additive to be efficaciously integrated into multiple small-to-large-scale sustainable transportation projects.

## Figures and Tables

**Table 1:** Physicochemical properties of base AP-5 asphalt cement and waste shoe polish (WSP).

Properties	Item	Base AP-5 Asphalt *(Mean ± SD)*	Waste Shoe Polish (WSP) *(Mean ± SD)*
Elemental Analysis	C (Carbon)	83.46 ± 0.05 wt. %	75.01 ± 0.43 wt. %
	H (Hydrogen)	9.95 ± 0.06 wt. %	12.52 ± 0.15 wt. %
	N (Nitrogen)	0.68 ± 0.21 wt. %	0.16 ± 0.01 wt. %
	S (Sulfur)	5.07 ± 0.30 wt. %	00.00 ± 0.00 wt. %
	O (Oxygen)	0.54 ± 0.04 wt. %	1.63 ± 0.03 wt. %
SARA Generic Fractions	Saturates	4.20 ± 0.50 wt. %	31.58 ± 1.27 wt. %
	Aromatics	19.20 ± 3.03 wt. %	00.00 ± 00.00 wt. %
	Resins	54.87 ± 3.38 wt. %	11.88 ± 1.76 wt. %
	Asphaltenes (-like components)	21.70 ± 1.44 wt. %	56.54 ± 0.67 wt. %
Volatile Organic Compounds	VOCs	Unspecified	2.80 ± 00.00 wt. % ^†^
Physical Characteristics	Penetration at 25 °C	59.16 ± 0.51 dmm	Unspecified
	Softening point	48.40 ± 00.00 °C	70.85 ± 0.17 °C
	Rotational viscosity at 135 °C	71.50 ± 00.92 cP	00.00 ± 00.00 cP
	Ductility at 25 °C	138.00 ± 1.15 cm	Unspecified
	Density at 25 °C	1.00 ± 00 g cm^–3^	0.80 ± 00 g cm^–3^
	Flash point	358 ± 00.00 °C	35.60 ± 00 °C
	Fire point	374.00 ± 00.00 °C	40.60 ± 00 °C
	Form	Highly viscous fluid	Paste
	Color	Black	Black
	Odor	Strong tarry	Solvent-like

**^†^** As stipulated by US Federal and State Consumer Product Regulations (Low VOCs).

**Table 2:** Basic formula of waste shoe polish (WSP) [40].

Ingredient	Chemical Formula	Technology
***Solvents***		
Hydrotreated light alkanes (Hydrotreated light petroleum distillates)	C13–C14 Alkane	-Carrier -Solubilizer -Preservative -Gelling agent
1-Chloro-4-trifluoromethylbenzene (PCBTF)	C_7_H_4_ClF_3_	-Carrier -Cleaning agent
Oleic acid	C_18_H_34_O_2_	-pH adjuster -Cleaning agent -Dispersant/Emollient
***Wax***		
***Natural wax***		
Carnauba wax (Brazil/Palm wax)	C_7_H_5_HgNO_3_	-Polishing agent -Waterproofing agent
Montan acid wax	C_30_H_60_O_3_	-Binder -Stabilizer -Viscosity-controller -Antistatic agent -Film-former
Glycol montanate	C_30_H_60_O_3_	-Emulsifying agent -Film-former -Polishing agent -Waterproofing agent
***Synthetic wax***		
Paraffin wax (Petroleum wax)	C_n_H_2n+2_	-Emollient -Thickener -Film-former -Waterproofing agent
Hydrocarbon waxes (petroleum), clay-treated microcrystalline	C_20_H_23_N_5_O_6_S	-Binding/Stiffening agent -Viscosity controlling agent -Emollient
***Plasticizers/Texturing agents***		
Polyethylene	(C_2_H_4_)_n_	-Waterproofing agent -Polishing agent -Film-former
Dimethicone (Silicon oil)	(C_2_H_6_OSi)_n_	-Emollient -Film-former -Waterproofing agent
Calcium stearate	C_36_H_70_CaO_4_	-Gelling agent -Thickener -Emulsion stabilizer
***Dyes/Pigments***		
C.I. Solvent Yellow 56 (C.I. 11021)	C_16_H_19_O_3_	-Coloring agent
C.I. Solvent Black 7 (C.I. 50415:1)	C_16_H_26_ClIN_2_OSi	-Coloring agent

**Table 3:** Vertical limit peaks with their respective functional groups [43].

Area	Vertical Peak Limit (cm^–1^)	Functional Groups
A_724_	734–710	Long chains
A_743_	783–734	Out of plane adjacent
A_814_	838–783	Out of plane adjacent
A_864_	912–838	Out of plane singlet
A_1030_	1047–995	Oxygenated functions—sulfoxide
A_1376_	1390–1350	Branched aliphatic structures
A_1460_	1525–1395	Aliphatic structures
A_1600_	1670–1535	Aromatic structures
A_1700_	1753–1660	Oxygenated functions—carbonyl
A_2862_	2880–2820	Stretching symmetric
A_2953_	2990–2880	Stretching aromatic

**Table 4:** Multiple stress creep recovery (MSCR) parameters attained at 0.1 kPa, 3.2 kPa, and 64 °C, for RTFO-aged base AP-5 asphalt and its specimens containing varying WSP percentages (e.g., 5, 10 and 15 wt. %).

Asphalt Binder Type	MSCR Data at 64 °C					
	*R*_0.1_ (%)	*R*_3.2_ (%)	*J_nr_*_0.1_ (kPa^–1^)	*J_nr_*_3.2_ (kPa^–1^)	∆ *J_nr_* (%)	PG Grade
AP-5 WSP 0 wt. %	–4.20	–8.00	1.2439	1.4237	14.50	PG 64 H
AP-5 WSP 5 wt. %	–4.80	–8.80	1.9205	2.1152	10.10	PG 64 S
AP-5 WSP 10 wt. %	–5.10	–9.10	2.5136	2.7994	11.40	PG 64 S
AP-5 WSP 15 wt. %	–4.30	–9.10	2.7724	3.2335	16.60	PG 64 S

## Data Availability

The data presented in this study are available on request from the corresponding author.
